# Biofilm viability checker: An open-source tool for automated biofilm viability analysis from confocal microscopy images

**DOI:** 10.1038/s41522-021-00214-7

**Published:** 2021-05-14

**Authors:** Sophie E. Mountcastle, Nina Vyas, Victor M. Villapun, Sophie C. Cox, Sara Jabbari, Rachel L. Sammons, Richard M. Shelton, A. Damien Walmsley, Sarah A. Kuehne

**Affiliations:** 1 EPSRC Centre for Doctoral Training in Physical Sciences for Health, University of Birmingham, Edgbaston, Birmingham UK; 2 School of Dentistry, University of Birmingham, 5 Mill Pool Way, Birmingham, UK; 3 School of Chemical Engineering, University of Birmingham, Edgbaston, Birmingham UK; 4 School of Mathematics, University of Birmingham, Edgbaston, Birmingham UK; 5 Institute for Microbiology and Infection, University of Birmingham, Edgbaston, Birmingham UK

**Keywords:** Biofilms, Biological techniques, Bacteria

## Abstract

Quantifying biofilm formation on surfaces is challenging because traditional microbiological methods, such as total colony-forming units (CFUs), often rely on manual counting. These are laborious, resource intensive techniques, more susceptible to human error. Confocal laser scanning microscopy (CLSM) is a high-resolution technique that allows 3D visualisation of biofilm architecture. In combination with a live/dead stain, it can be used to quantify biofilm viability on both transparent and opaque surfaces. However, there is little consensus on the appropriate methodology to apply in confocal micrograph processing. In this study, we report the development of an image analysis approach to repeatably quantify biofilm viability and surface coverage. We also demonstrate its use for a range of bacterial species and translational applications. This protocol has been created with ease of use and accessibility in mind, to enable researchers who do not specialise in computational techniques to be confident in applying these methods to analyse biofilm micrographs. Furthermore, the simplicity of the method enables the user to adapt it for their bespoke needs. Validation experiments demonstrate the automated analysis is robust and accurate across a range of bacterial species and an improvement on traditional microbiological analysis. Furthermore, application to translational case studies show the automated method is a reliable measurement of biomass and cell viability. This approach will ensure image analysis is an accessible option for those in the microbiology and biomaterials field, improve current detection approaches and ultimately support the development of novel strategies for preventing biofilm formation by ensuring comparability across studies.

## Introduction

Biofilms are defined as ‘aggregates of microorganisms in which cells are embedded in a self-produced matrix of extracellular substances that are adherent to a surface'^1^. Compared with planktonic bacteria, those present in biofilms can survive harsher environments and demonstrate increased resistance to antimicrobials^2^. Biofilms account for up to 80% of implant-related infections as, unintentionally, medical implants provide excellent surfaces for formation of these 3D bacterial communities^3^. Device-related infections are particularly difficult to eradicate and often result in the need for restorative surgeries^4^. Furthermore, there is also an increased concern regarding the presence and spread of antimicrobial-resistant strains in biofilms^5^. It is therefore vital to consider these complex structures when evaluating antimicrobial activity in the development of functional biomaterials and new antibacterial approaches to tackle device-related infections.

To investigate the effect of novel antimicrobials and surface functionalisation, quantification of biofilm development and viability following such treatment is essential. Traditionally in microbiology, analysis of biofilms is performed through serial dilution of a culture to count the number of colony-forming units (CFUs), or alternatively using crystal violet stain along with spectrophotometry^6–10^. Whilst these traditional methods have their applications and advantages, a move towards more direct quantitative analyses of biofilms that reduce operator variability is recommended. Furthermore, neither CFU-plating nor crystal violet staining allow for detailed visualisation of biofilm architecture. Understanding 3D structure is important because extracellular polymeric substances (EPS) can contribute to antimicrobial resistance properties of biofilms by impeding transport of some antibiotics^11,12^. Disruption of biofilm architecture to expose cells and increase the efficacy of antimicrobial drugs is a potential approach to tackle device-related infections, and therefore is an important aspect to consider^13^.

In contrast, direct imaging of biofilms using microscopy techniques provides information on their structural characteristics, which can in turn determine whether an intervention has been successful in disrupting biofilm formation. Confocal laser scanning microscopy (CLSM) selectively excites fluorescence signals from different planes within a sample, acquiring images point by point with localised laser excitation at specific wavelengths. CLSM is a useful technique as it enables 3D visualisation of biofilm structure by excluding signals from adjacent planes. A second benefit of CLSM is the versatility offered by fluorescent stains added to a sample, allowing further information to be obtained; for example, the presence of extracellular DNA, exopolysaccharides and biofilm viability. CLSM with viability staining provides high sensitivity, specificity and resolution^14^. Of the fluorescent stain protocols available, live/dead staining is a conventional method of evaluating biofilm formation in microbiology for a wide variety of applications including oral, bone and gut microbes^15–20^. A live/dead stain provides a fluorescence assay of bacterial viability, based on membrane integrity. Most commonly, SYTO® 9 acts as the green fluorescent nucleic acid stain, labelling bacteria with intact cell membranes, and propidium iodide forms the red-fluorescent nucleic acid stain, penetrating only bacteria with damaged membranes^21^. Examples of the application of CLSM and fluorescent staining to biofilms include examining *Pseudomonas aeruginosa* (*P. aeruginosa*) biofilm formation on antibiotic-loaded bone cement^19^, observing the effect of antimicrobial therapy on biofilm formation in endotracheal tubes^18^ and screening cinchona alkaloids for anti-biofilm activity against *Staphylococcus aureus* (*S. aureus*)^22^.

Despite the varied and wide-ranging use of CLSM and live/dead staining to investigate biofilm formation, there is little consensus regarding evaluation of the resulting micrographs. Specifically, there is no consistent method applied for quantifying live/dead bacteria from the confocal images reported in the literature. Some groups use CLSM to simply visualise the biofilm and qualitatively interpret the results, or conduct manual segmentation by using a global threshold or delineating the cells in the images manually^16,23,24^. Simple segmentation methods such as these are time consuming and may result in inconsistencies due to user subjectivity. Other studies elect not to report in full their chosen segmentation algorithm or validate its accuracy^19,25–27^. One useful way of validating accuracy is to perform a sensitivity and specificity analysis that determines whether an algorithm can successfully detect a pixel that corresponds with bacteria and a pixel that corresponds with background, respectively^28^. While more robust segmentation protocols have been reported, they are not always accessible or reproducible if the method lacks detail and they may be particularly challenging to implement for non-experts. Many studies use bespoke software such as Imaris^25^, COMSTAT^29^, PHLIP^30^ and most recently BiofilmQ^31^. These can make CLSM micrograph analysis easier to navigate through a user-interface. BiofilmQ can measure features from biofilm images to extract information such as fluorescence intensity, biofilm density and surface area. However, it is not specifically developed for cell viability measurements of biofilms, and currently there is no option for morphological operations, which were used in the macro developed in the current study. Whilst the algorithms used in some bespoke software are made available, an understanding of the settings in each package and how these impact on the data is required. These settings should be reported for a study to be repeated. Furthermore, it is necessary to report any image pre-processing as this will affect comparability across literature.

In addition to navigating the range of segmentation methods and software available, the commonly used stain for bacterial biofilm viability, the FilmTracer™ LIVE/DEAD® Biofilm Viability Kit (Invitrogen, USA)^21^, can give erroneous results if images are not analysed correctly. Depending on the contrast of the red and green channel images, bacteria which are dead can appear yellow in images (due to red and green being superimposed on each other)^32,33^. A further challenge with the FilmTracer™ LIVE/DEAD® Bacterial and Biofilm Viability Kits is that propidium iodide can stain extracellular DNA that is present in biofilms^34^. Therefore, qualitative observation of live/dead stained biofilms could lead to misleading conclusions since the contrast of each channel is manually adjusted by the user. If automated image analysis is used to analyse the red and green channels separately this would give more objective quantitation with no possibility of the two channels being superimposed. Although numerous studies have published new image analysis techniques for biofilms^35–39^, many microbiological studies that use image processing still do not report the exact methods used, including the type of threshold applied. Such information is critical to determining accuracy of the study and ensuring reproducibility. Ultimately this leads to the conclusion that the current suite of image processing tools available for biofilm analysis is difficult to access and cumbersome for non-specialists with no significant programming experience. This highlights a gap for an open-source image analysis tool designed specifically to assess biofilms which balances accessibility, transparency and accuracy.

This work aims to develop a robust but easy to use automated image analysis technique to quantify biofilm formation from confocal micrographs, which accounts for the errors identified with SYTO® 9 and propidium iodide stains. A new image analysis method is proposed that incorporates image pre-processing and automated thresholding, using the open-access software Fiji (ImageJ, US National Institutes of Health, Bethesda, Maryland, USA). To the authors’ knowledge, no prior studies have directly compared the results of confocal micrograph image analysis with those of counting CFUs and therefore this was undertaken in the present work. Alongside method comparison, sensitivity and specificity of the automated image analysis was carried out to evaluate its accuracy. Further validation of the method was conducted on Gram-positive and Gram-negative species of different cell morphologies: *P. aeruginosa*, *Lactobacillus casei*, and a multi-species biofilm consisting of *Fusobacterium nucleatum, Actinomyces naeslundii, Streptococcus gordonii* and *Porphyromonas gingivalis*. A unique aspect of this work is the use of translationally relevant case studies to trial the automated image segmentation protocol, the results of which will also be presented. This analysis method will prove useful by ensuring reproducibility across studies, by offering a faster analysis approach than traditional microbiological methods enabling higher sample numbers, and finally by reducing human error compared with CFU-counting or manual image segmentation. Ultimately, this work will support the development of much needed approaches to prevent and treat costly infections.

## Results

### Validation of image analysis protocol

**Fig. 1:**
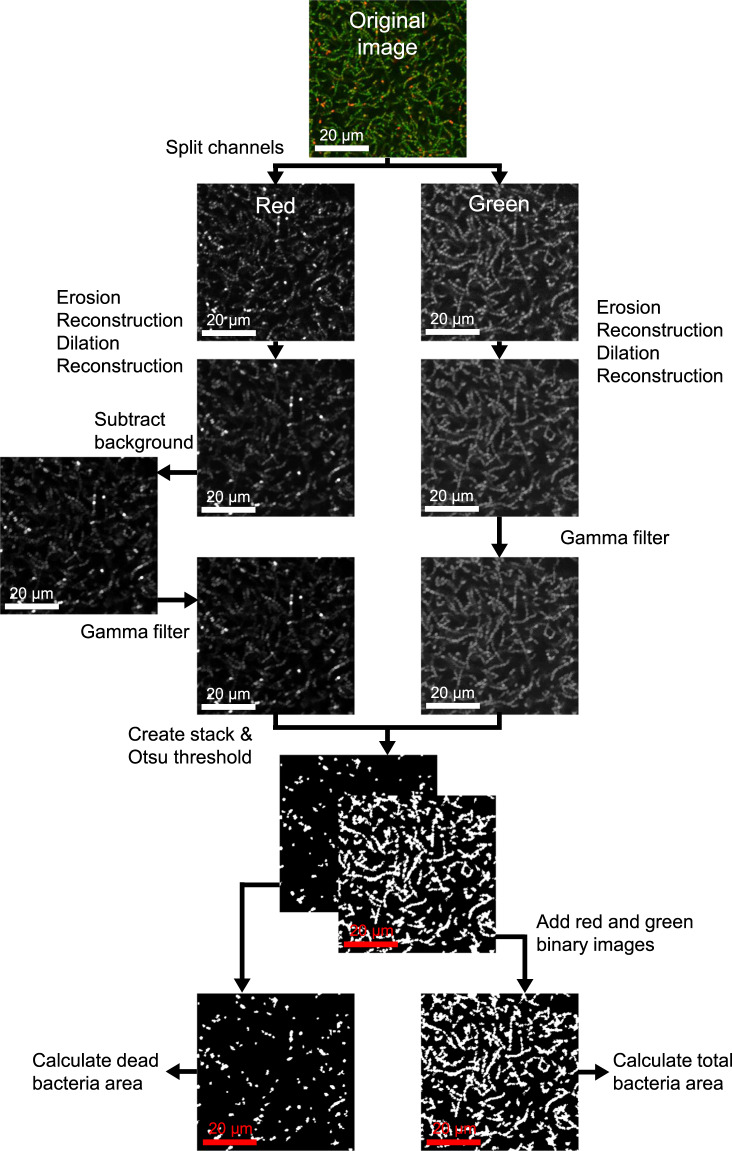
Image analysis steps used in ImageJ to calculate bacterial viability from a confocal image of biofilm with LIVE/DEAD stain. Images taken from a representative *S. sanguinis* biofilm cultured for 48 h (20 µm scale bar). See Supplementary Information to implement the automated analysis.

**Fig. 2:**
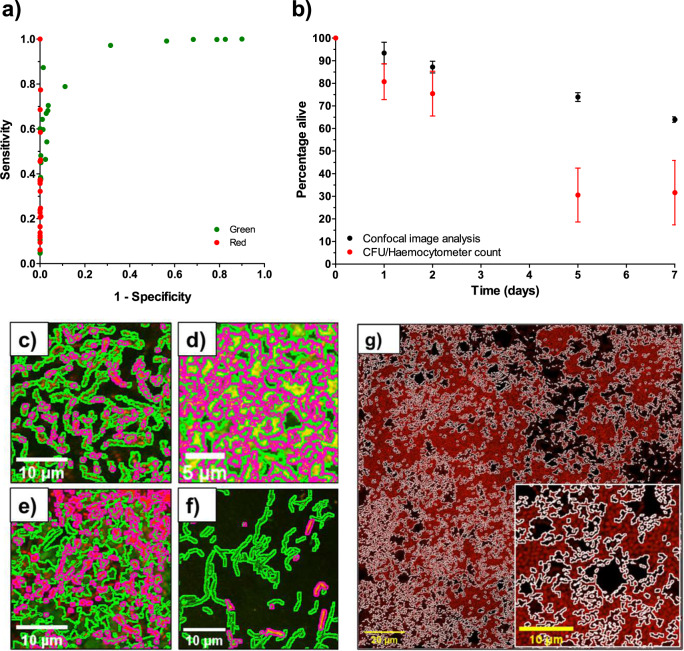
Results of validation of automatic image analysis protocol. **a** ROC curve demonstrating sensitivity and specificity of the image analysis protocol. Green points represent sensitivity and specificity of the green channel (total cells) and red points represent sensitivity and specificity of the red channel (dead cells). **b** Comparison of image analysis and biological methods. Figure shows mean ± standard deviation (for image analysis, five confocal images were analysed of each of five biological replicates, *N* = 5 and for biological methods, three biological replicates were analysed, *N* = 3). To obtain the percentage viability using biological methods, live cells were counted using a serial dilution and CFU-plating. Total cell count was obtained using a haemocytometer. **c**–**f** Sample images of a variety of single-species biofilms demonstrating result of automated image analysis. The green outline indicates the total bacteria area and the magenta outline indicates the dead bacteria area. **c** *S. sanguinis* (10 µm scale bar), **d** *P. aeruginosa* (5 µm scale bar), **e** multi-species biofilm consisting of *F. nucleatum*, *A. naeslundii*, *S. gordonii* and *P. gingivalis* (10 µm scale bar), **f** *L. casei* (10 µm scale bar). **g** Representative micrograph of an *S. sanguinis* biofilm treated with 5% CPC to demonstrate the ability of the macro to handle extreme conditions (Full image 20 µm scale bar, small image 10 µm scale bar). The magenta line shows the result of the segmentation of the red channel. The resulting output from the macro is 0% viability.

To assess the reliability and accuracy of the automated protocol developed (Fig. 1), a series of analyses were performed. This included sensitivity and specificity analysis^28^, a comparison with traditional microbiological techniques and the application of the protocol to a variety of bacterial species with varying morphologies (Fig. 2).

The sensitivity and specificity of the image analysis method was determined using receiver-operating characteristic (ROC) analysis (Fig. 2a). A ROC curve is a plot of sensitivity (true positive rate) versus 1 – specificity (false positive rate). The greater the algorithm’s ability to correctly identify pixels in an image, the closer the curve sits to the upper left-hand corner of the graph^40^. A ROC curve lying on the diagonal reflects a performance that is no better than identifying pixels by chance. The ROC analysis in the present study demonstrated that the specificity for both red and green channels was high, with means of 99.9 and 81.7%, respectively. However, the sensitivity of the automated image analysis method in the red channel varied, ranging from 6.1 to 100.0%.

Figure 2b shows the resulting quantification of *Streptococcus sanguinis* (*S. sanguinis*) biofilm over time using the automated image analysis method developed in this work and CFU-plating combined with counting using a haemocytometer. Both methods demonstrated viability decreased with biofilm age, however, the rate at which this occurred varied significantly between the two methods. It should be noted that the traditional methods induced greater errors, with a coefficient of variation (CV) ranging from 17.0 to 78.1%, compared with 4.24 to 11.5% for image analysis, and this was likely due to the manual nature of the method. Manual analysis of CFU plating and cell counts using a haemocytometer typically result in wider errors due to the subjectivity of the user defining what is considered a cell, and from volume and dilution errors.

To confirm that the developed analysis could be performed on biofilms of species with different morphologies, the ImageJ macro was applied to 24-h *P. aeruginosa* and 7-day *L. casei* biofilms, and to further challenge it, a 5-day multi-species biofilm consisting of *F. nucleatum ssp polymorphum*, *A. naeslundii*, *S. gordonii* and *P. gingivalis* (Fig. 2c–f). *L. casei* and *P. aeruginosa* were selected due to their rod-shaped cell morphologies, to contrast with the cocci-shaped *S. sanguinis*. The protocol was applied to a multi-species biofilm containing a range of morphologies to ensure it could accurately determine biofilm viability and coverage in more challenging and complex images. Figure 2c–f show that the analysis protocol successfully identified live and dead bacteria of different morphologies. Through qualitative observation of the outline of stained bacteria (Fig. 2c–f), this was evidenced by very few bacteria being incorrectly identified as background by the automated segmentation method.

It is important to ensure that any image analysis method can cope with a wide range of conditions. In the development of antimicrobial techniques and novel implant surface coatings, it is expected that conditions which include no viable cells in biofilms will be analysed. To ensure that the protocol handles such conditions, the macro was applied to *S. sanguinis* biofilms treated with the antimicrobial cetylpyridinium chloride (CPC) at bactericidal levels (Fig. 2g). The macro consistently produced results of 0% alive (*n* = 6) for all biofilms treated with CPC. This confirmed it was reliable across a range of biofilm viabilities.

### Translation of image analysis method to research applications

The aim of the present research was to develop an accurate image analysis protocol that will aid in the development of novel antimicrobial therapies and implant devices. To investigate the potential of this protocol, it was applied in three key experiments. First, to demonstrate it could provide useful data to examine the effectiveness of antimicrobial compounds, a simple mouthwash study was performed. In this experiment, a commercial mouthwash was applied to biofilms of two species: *P. aeruginosa*, a pathogen which is known to have increased antibiotic resistance^41^, and the commensal oral bacteria, *S. sanguinis*^42^. Secondly, biofilms of *Staphylococcus epidermidis* (*S. epidermidis*) were grown on additively manufactured (AM) Ti-6Al-4V coupons to understand the effect of manufacturing protocols on the viability of a frequently detected pathogen in implant infections^43,44^. Finally, to demonstrate the information that can be obtained regarding the 3D architecture of biofilms, the automated protocol was applied to z-stacks taken from 1-day- and 7-day-old *S. sanguinis* biofilms, the results of which are described below.

**Fig. 3:**
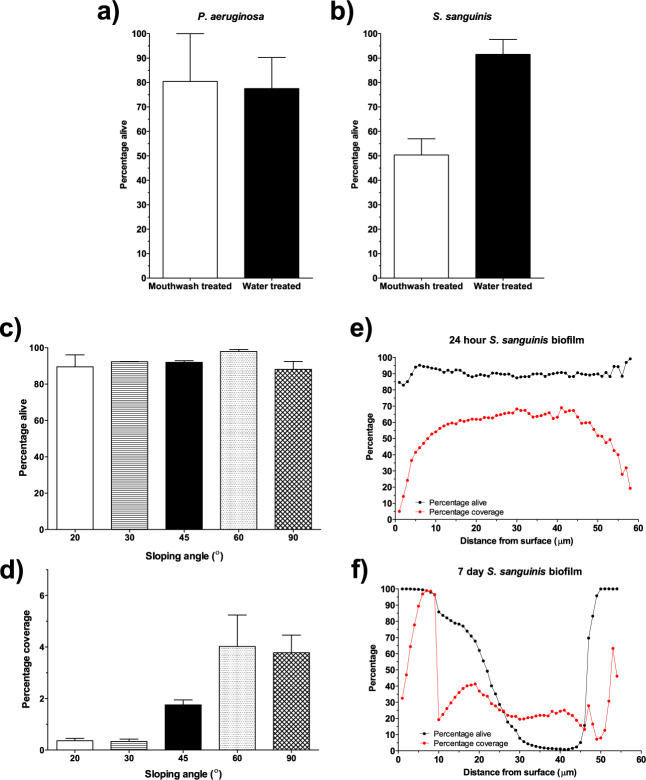
Translation of image analysis method to research applications. **a**, **b** Simple mouthwash study comparing biofilms of **a** *P. aeruginosa* and **B** *S.* *sanguinis* treated with mouthwash or water (*n* = 3 for all conditions). **c**, **d** Analysis of *S. epidermidis* biofilms grown on additively manufactured coupons at different sloping angles: **c** Percentage alive and **d** Percentage coverage. **e**, **f** Biofilm coverage and viability with increasing distance from coverslip for **e** a 24-h biofilm of *S. sanguinis* and **f** 7-day biofilm of *S. sanguinis*. Z-stacks were taken at 1 µm increments from the surface (the first plane in which bacteria were identified), and hence the distance from the surface is equivalent to the biofilm thickness.

Applying mouthwash to biofilms of two different species showcased that mouthwash had a limited effect on the biofilms of *P. aeruginosa* when compared with water-treated samples (*p* = 0.93) (Fig. 3a). Whilst there was no significant difference identified between the mouthwash-treated and water-treated biofilms of *S. sanguinis*, the *p* value was much lower (*p* = 0.08) (Fig. 3b). It is expected that a larger study would support the effectiveness of the mouthwash treatment at reducing the percentage of live bacteria in *S. sanguinis* biofilms, as evidenced by other literature^45,46^. This was not the objective of the present study, rather the aim was to demonstrate the application of the automated image analysis. The findings of this experiment agreed with other work that has highlighted the resistance of *P. aeruginosa* to a range of broad-spectrum antibiotics found in commercial mouthwashes^47,48^.

A second area of research that is highly important in the field of antimicrobial resistance is the development of novel materials, coatings, and surface treatments for medical and dental implants. To showcase the applicability of the current approach in the development of novel medical devices, two properties of *S. epidermidis* biofilms on AM titanium implants manufactured with different orientations (20° to 90° from the normal plane, see Supplementary Fig. 2) were investigated, namely cell viability and coverage (Fig. 3c, d). Orientation of AM samples significantly modified the resulting average roughness from 8 µm up to 18 µm, for 20° and 90° respectively, as shown in previous works^44^. Nevertheless, the number of live bacteria expressed as a percentage of total bacteria showed no significant difference (*p* = 0.07) between surfaces (Fig. 3c). In contrast, when percentage coverage was analysed (as it relates to biomass), it demonstrated that increasing the sloping angle resulted in a significant increase in percentage coverage of the biofilm (*p* = 0.02) (Fig. 3d). The difference between viability and coverage indicated that albeit an increase in biomass developed on the surface of the samples, the percentage of living cells is not dependent on surface modification. This could be the result of the selected alloy lacking any antimicrobial effect^49^, coupled with the larger surface area and shear force protection offered by the peaks and valleys present in the rougher samples, leading to more favourable growth conditions^50,51^.

The analysis protocol can be used to investigate biofilm composition from coverslip to biofilm surface by applying it to each image of a confocal z-stack. In addition, the total number of pixels that correspond with bacteria (live or dead) can also be used to calculate biomass as ‘percentage coverage’, i.e. the number of stained pixels as a percentage of total pixels in the image. This was carried out for an *S. sanguinis* biofilm cultured for 1 and 7 days on a Thermanox coverslip (Fig. 3e, f, respectively). For the 24-h biofilm, viability remained consistent throughout, ranging between 82.9 and 99.18%. However, the coverage increased in the centre of the biofilm, peaking at 69.0% at 41 µm from the coverslip, and decreased towards the surface, ending at 19.24% at 58 µm from the coverslip. In contrast, viability varied significantly across the 7-day biofilm. Low viability was observed in the centre, with values lower than 50% for distances between 23 to 46 µm from the coverslip surface. In contrast, high viability was detected at the coverslip interface (99%) and on the biofilm surface (100%). The reduction in viability in the centre of the biofilm may be due to nutrients being limited and unable to reach those species in the centre or could be caused by an oxygen gradient throughout the biofilm. Percentage coverage, which relates to biomass, also decreased in the centre of the older biofilm, dropping below 45% between 10 and 52 µm from the coverslip, which may have been linked to the fact that viability had decreased significantly.

## Discussion

The aim of this study was to develop a method to analyse confocal micrographs of live/dead stained biofilms. The method was designed to be simple and accessible to a range of researchers that work on the development of antimicrobial strategies related to biofilms, including microbiologists and materials scientists. Therefore, the analysis protocol was written in the open-source software ImageJ and the method requires no preliminary image preparation or modification. The ImageJ macro, alongside a fully detailed description of how to execute the algorithm to enable researchers to implement this protocol, is available in Fig. 1 and the Supplementary Information. The algorithm was effective and accurate on a range of biofilms, including different bacterial morphologies, such as cocci (*S. sanguinis*) and rod-shaped (*L. casei*) bacteria, and different biofilm ages (from 24 h up to 7 days). A full workflow is provided alongside a series of validation methods in this study, and furthermore includes application of the code to translational case studies, which is an advantage compared with other literature that include analysis of CLSM micrographs of biofilms.

Over the past two decades, many researchers have attempted to tackle the problem of automated analysis of biofilm micrographs, including developing commercial and free software tools^29–31^. Depending on the size of the cells, the quality and resolution of the image, and thickness or density of the biofilm, two approaches to automated quantification can be taken: (1) by detecting and counting discrete objects or cells or (2) by making assumptions regarding the manner in which the properties of the entire image relate to the biofilm characteristics. In this study, the latter approach was taken, and the number of pixels stained red and green were used to quantify the number of viable (live) cells as a percentage of total cells. The reason for selecting this method was driven by the small cell size and common overlapping of bacteria in biofilm images, even in high-resolution CLSM micrographs. Furthermore, detecting discrete cells can be challenging if they have different morphologies. Therefore, making assumptions about the relationship between pixel count and bacteria viability ensured that the automated approach was accurate and applicable to a range of cell morphologies. The total number of pixels that correspond with bacteria (live or dead) can also be used to calculate biomass as ‘percentage coverage’, i.e. the number of stained pixels as a percentage of total pixels in the image.

A number of studies that utilise CLSM to analyse biofilm formation simply visualise the biofilm and report qualitative results, or conduct manual segmentation of the micrographs^16,23,24^. The challenge with these approaches makes comparison with other literature difficult due to the subjective data. Neither of these methods take into consideration any non-specific staining or extracellular matrix staining that may occur when using the FilmTracer™ LIVE/DEAD® Biofilm Viability Kit^34^. Furthermore, segmentation methods that involve manually selecting the cells in an image are time consuming and may result in inconsistent segmentation. To address these challenges, the image analysis protocol presented in this study demonstrated consistent and repeatable results. This was evidenced by a small standard deviation with a CV between 4.24 and 11.5% in the large biofilm study with over 100 images of 20 biofilms (Fig. 2b).

A further limitation of studies that implement CLSM micrograph analysis is that many elect not to report in full the chosen segmentation algorithm or validate its accuracy, negatively influencing reproducibility and comparison^19,25–27,52,53^. It is vital that the validity of an image segmentation algorithm is demonstrated across different species, on ‘extreme’ cases such as all-dead biofilms, and by comparing with separate techniques. In this study we carried out a series of validation steps to demonstrate that the protocol was effective and accurate. This included performing a sensitivity and specificity analysis to compare the automated results with manual segmentation of the images. This manual segmentation represented a ‘ground truth’ state, although it should be noted that the manual segmentation was conducted on the original images with no pre-processing and therefore background fluorescence and extracellular matrix staining had not been accounted for. The ROC study showed good sensitivity and specificity for the green channel (total bacteria), 60.3 and 81.7%, respectively, and very good specificity of 99.9% for the red channel (dead bacteria), although the sensitivity for the red channel had a wider range (Fig. 2a). This was likely to arise from the additional steps implemented to remove background noise in the red channel necessary to prevent the analysis from including red areas that were not bacteria. Reduced sensitivity in the red channel was needed due to the challenge of extracellular matrix staining that occurred and therefore the potential to underestimate the percentage of live cells^32–34^. Furthermore, the ROC curve was calculated by comparing the resulting binary images after running the automated analysis with manually segmented sections of images (determined by manually delineating all bacteria) that underwent no pre-processing. During manual segmentation it is likely that low-level background fluorescence combined with the red propidium iodide stain marking EPS, results in the ‘ground truth’ images used to calculate the ROC curve including pixels that are not bacteria. This also contributes to the lower sensitivity of the automated analysis in the red channel.

Very few prior studies have directly compared image analysis with traditional quantification methods. As an example of those that have, Larimer et al. (2016) compared the cell coverage determined by image analysis with cell coverage determined by measuring the optical density of the biofilm^39^. In the current study we compared cell viability determined using image analysis with that determined using CFU-plating and cell counting, which helped to build confidence in the presented approach. Figure 2b demonstrates that the overall trend in live cell percentage varied between the image analysis and manual counting methods. Using traditional techniques such as CFU-plating and counting cells using a haemocytometer resulted in larger errors as the age of the biofilm increased, rising from a CV of 17.0% at 24 h to a CV of 78.1% at 7 days. It also lead to a lower percentage of live cells at later time points compared with the automated image analysis data; for example, the mean percentage alive at 7 days calculated by image analysis was 63.9%, whereas from traditional techniques it was determined to be only 31.6%. This could have been due to the increased number of cells present in the larger, older biofilms, making counting the cells manually less accurate. Figure 2b suggests that automated image analysis was likely to be more accurate and therefore a better method to identify statistically significant variations between biofilm growth conditions when researching antimicrobial approaches. Other benefits to the image analysis method presented are detailed in Table 1.

**Table 1:** Summary of advantages and disadvantages of image analysis and CFU-plating combined with counting cell in a haemocytometer to quantify biofilm formation.

Method	Approach	Advantages	Disadvantages
Fiji macro (ImageJ)	▪ Correct for uneven fluorescence intensities ▪ Remove noise ▪ Segment bacteria from background using Otsu threshold ▪ Record number of pixels for total bacteria ▪ Apply same process for red channel only to determine dead bacteria count.	✓ Open-source software ✓ Can run macro on multiple images at once ✓ Time taken to run image analysis on 25 CLSM micrographs is <10 min.	✗ Requires some data manipulation after running the automated segmentation to calculate biofilm viability from pixel count. ✗ Workflow may need altering to observe larger mammalian cells or for alternative staining protocols.
Biological methods	▪ Determine number of live cells from CFU-plating. ▪ Determine total cell number using haemocytometer.	✓ No specific software required ✓ Actual cell number determined rather than inferred from pixel number.	✗ Time-consuming ✗ Resource-intensive ✗ Susceptible to human error ✗ Challenging for larger, increased density biofilms as further dilution required to analyse.

Some of the more frequently used software packages developed specifically for biofilm analysis, such as COMSTAT and BiofilmQ, have an easy to use graphical user interface that make them popular for use. However, one main drawback of both of these packages is that they do not have options to apply filters and morphological operators as with those used in the current study^29,31^, which allow for accurate detection of the bacteria in the image whilst neglecting EPS and non-specific staining, commonly found in biofilms. In addition, these software packages rely on the user deciding if pre-processing of the images is necessary, deciding which operators to apply and implementing any pre-processing, which is difficult for those with no prior knowledge of image analysis. In the present work, morphological operators and filters were included in the automated protocol to remove background fluorescence and account for potential staining of the extracellular matrix or e-DNA, particularly in the red channel. Furthermore, ImageJ is open-source and familiar to many researchers. Presenting the full macro created in this study (see Supplementary Information) enables users to adjust the gamma values, structuring element size and add or remove steps in the image pre-processing according to their data.

Numerous studies have been published that develop new image analysis methods for biofilm micrographs, however, many present several hurdles before they can be applied by non-specialists. For example, they are often created in proprietary software such as MATLAB^35–37^ or in programming languages such as C + + ^38^, which make them difficult to use for researchers with little or no programming experience. In some studies, the chosen image segmentation technique was applied to low resolution images where individual bacteria were not visible^39^. In the present research, high resolution (x40 magnification, numerical aperture 1.30) images were used to ensure the segmentations were accurate. Some published studies that use open-source software have not included the code to allow for easy replication by other scientists wishing to use their method. One of the key strengths of this work is that a copy of the code, instructions on how to implement it and an overview of the image analysis protocol are all provided to ensure reproducibility (see Fig. 1 and Supplementary Information)^14^. This allows for users to understand the code and easily modify it to fit the data being analysed. For example, if a different staining protocol is used, the pre-processing steps can be removed or adjusted so as not to account for the issues identified with the FilmTracer™ LIVE/DEAD® Biofilm Viability Kit. A further strength of the protocol presented is that it has low computational time, with 25 micrographs analysed in less than 10 min (Table 1). This allows for an increased number of samples to be analysed and can ultimately improve the robustness of studies investigating antimicrobial techniques to reduce implant-related infection.

There are, however, several limitations to using a method based on CLSM micrographs. Firstly, it is not possible to evaluate the entire biofilm at once; in this study imaging was performed at x40 magnification to obtain high resolution images of individual bacteria in the biofilm. Averaging data from across the biofilm sample and increasing the number of repeats can limit the impact of this. In this work, five images were taken across five samples for each biofilm condition in Fig. 2b. As the analysis protocol in ImageJ can process many images quickly, increasing the number of samples to account for the limited range of the confocal images was straightforward. Linked to this, a second limitation of the work presented its application to poorer quality micrographs. For example, if a sample is not completely flat when imaged using the CLSM, an area of the image may be over or under exposed and this can affect the resulting analysis. It is advisable to take appropriate steps to ensure optimal imaging of the samples. These include ensuring the fluorescent dye has sufficient signal to avoid noise caused by increasing the contrast artificially, ensuring the sample stays horizontal during sample preparation and imaging using a high numerical aperture/magnification to obtain high resolution images. Individual bacteria should be visible in the micrographs being analysed and it is recommended that a minimum magnification of x40 be used to implement the described method. It should also be noted that the results of the analysis will be more subjective if the user selects the location on the biofilm for the image to be taken. User subjectivity can be reduced significantly by taking a high number of images at random locations across the biofilm; a minimum of five per sample is recommended. A final challenge where this workflow demonstrated limitations was that the macro had been tailored specifically for bacterial biofilms and for fluorescent images that were stained with the FilmTracer™ LIVE/DEAD® Biofilm Viability Kit. For this reason, the additional steps taken to reduce the error caused by SYTO® 9 would affect the results if a different fluorescent stain is used by over-reporting viability. Whilst there is potential for the macro to be applied to other confocal images, the workflow may need altering to examine larger mammalian cells or alternative staining protocols. However, this should be possible for users with some image analysis experience, as each step of the macro has been described within the code.

Despite the limitations of the proposed approach, it is important to reiterate that CLSM and automated micrograph analysis can prove very useful for researchers working on antimicrobial strategies. The study of antimicrobial strategies to tackle device-related infections is a vital area of research due to the global challenge of antimicrobial resistance^54,55^. Comprehensive efforts are needed to minimise the pace of resistance by studying novel antimicrobial agents and much research is being conducted to develop novel antimicrobial techniques^56^. Current reported image analysis methods for CSLM images of biofilms do not often demonstrate their application to a range of translational research. In the present work, the protocol was applied to three key areas that can benefit from automated CLSM micrograph analysis. The effect of antimicrobial compounds being developed on biofilms is highly important, given the increased resistance shown by bacteria in these complex 3D environments, as well as the knowledge that most bacteria exist in biofilm communities^57^. This is particularly crucial in the oral field, where broad-spectrum antibiotics are used in consumer products, such as toothpaste and mouthwash (e.g. chlorhexidine and CPC) and in the clinic to treat infection (e.g. amoxicillin and metronidazole)^58–61^. Applying the image analysis protocol to a small study on commercial mouthwash demonstrated that *P. aeruginosa* was resistant to the mouthwash. Studies that have previously reported the effect of broad-spectrum antimicrobials on oral pathogens typically identify the minimum inhibitory concentration (MIC) using a CFU-plating technique, measure optical density or report zones of inhibition^47,60^. However, these methods rely on individual interpretation so may be subjective, provide limited information on cell viability and typically result in high standard deviations for small sample numbers. Utilising the proposed analysis protocol in research to investigate new antimicrobial compounds would be effective at identifying potential novel therapeutics. It has benefits over traditional techniques as it produced low error from small sample numbers; in the present study the mean CV was 26.2% from *n* = 3. Furthermore, automated segmentation would ensure reproducibility and comparability across the literature.

A second area of research where preventing infection is paramount in implants and medical devices. Infection of implants can result in costly restorative surgeries and can also increase the failure rate of subsequent implant placement^62^. A specific example comes in the form of AM or bespoke implantable devices. These technologies are capable of producing personalised complex geometries while introducing features to enhance osseointegration (a structural and functional connection with the natural bone), reduce stress shielding and incorporate therapeutically loaded materials^63–65^. Nevertheless, clinical cases requiring such devices are commonly associated with complex interventions, typically arising from traumatic injuries, which may significantly raise infection rates by up to 23–40% for personalised cranioplasties^66,67^. Thus, much research is being conducted to reduce the occurrences of biofilm-related implant infections. One strategy to limit colonisation and proliferation of bacterial species results from careful selection of surface finish for AM materials to ensure the implant allows for osseointegration but prevents biofilm formation^44^. Villapún et al. (2020) demonstrated that in situ roughness control can be achieved through changing the orientation at which an implant is manufactured, with maximum mammalian cell adhesion and minimum *S. epidermidis* growth for printing angles between 20° to 30° to the normal plane^44^. To further assess the applicability of the CSLM image analysis protocol, Ti-6Al-4V coupons were AM and *S. epidermidis* biofilms were grown on the coupons^44^. The resulting biofilms were stained, imaged and two properties investigated through the image analysis protocol, namely viability (percentage alive) and biomass (percentage coverage), Fig. 3c, d, respectively. The percentage of live cells showed no significant changes with sloping angle modification; however, it was determined that an increase in sloping angle resulted in a rise in biomass for angles higher than 30°. This indicated that the growth of *S. epidermidis* biofilm was constrained, however there was no potential antimicrobial effect enacted from the metallic surface. The rise in biomass concurred with crystal violet and confocal image results reported by Villapún et al. (2020), while the lack of contact killing was expected from a bacteriostatic alloy such as Ti-6Al-4V^44^. Crystal violet staining can complicate the analysis of biofilm formation and potentially introduce artefacts during the recovery of the dye^68^. In contrast, confocal imaging is a more versatile method that can quantify biofilm viability and biomass accurately. The current automated method allows for subjectivity to be removed when interpreting CLSM micrographs and can generate additional information regarding cell viability when compared with crystal violet staining methods. Whilst viability did not change with surface roughness in this experiment, viability is a key parameter to obtain in future studies of this nature, where surface functionalisation may induce a bactericidal response that would not be picked up from crystal violet staining alone.

Finally, the translation of the presented method has a further application investigated in this research. One of the advantages of CLSM imaging is that it can generate an understanding of the 3D structure of a biofilm using z-stacks. Not only can this provide information about biofilm thickness and biomass, but the application of the image analysis protocol can elucidate information about biofilm composition throughout. Figure 3e, f show the viability and percentage coverage of an image stack taken of 1-day-old and 7-day-old *S. sanguinis* biofilms. For the younger biofilm, the percentage of live cells remained consistent and above 80% throughout its depth. However, in comparison the viability of the 7-day-old biofilm was reduced significantly in the centre and increased towards the surface. This could have been due to limited nutrients reaching the centre of the biofilm, combined with an oxygen gradient that increased towards the surface, thus resulting in cell death. The reduced coverage identified in the centre of the 7-day biofilm compared with the 24-h biofilm could be explained due to biofilm age. More mature biofilms that have increased EPS compared to early-stage biofilms may prevent the live/dead stain diffusing through to the centre, and this may explain the reduced coverage at the centre of the biofilms. Gaining an insight into the 3D structure of a biofilm, combined with information on viability, can enhance the understanding of the effect of antimicrobial compounds and materials. CLSM is an optimal tool for this as it has a large vertical range that can image a biofilm of up to 60 µm thickness, and fluorescence staining can provide information on viability. Applying the automated method described in this study to biofilms grown on modified surfaces could provide further information on how the modification is affecting the biofilm structure throughout. Gaining an understanding of biofilm composition is especially important when studying implant-related infections. This method could be applied to biofilms formed on modified implant surfaces to quantify antimicrobial effects. The advantage of the proposed segmentation method is that multiple images can be analysed very quickly and consistently, as well as ensuring each image within a single z-stack is treated the same, increasing comparability across samples.

In summary, this paper presents an image analysis protocol for quantifying CLSM micrographs of live/dead stained biofilms. The protocol was validated by comparing with other methods and on different species, and its use as an adjunct to traditional microbiology techniques was demonstrated, for example to support results from semi-quantitative methods such as crystal violet staining. Importantly, the method can be translated to antimicrobial drug and surface modification testing in many different industries and research fields. The key advantages of this protocol are that it is written in open-source software, is easy to use, transparent in function and is modifiable unlike other available software. This makes it a useful tool for those with different research backgrounds to enable quantitative analysis of biofilm viability to be performed. It has been demonstrated that the current approach is a reliable measurement of biofilm growth and cell viability assessment, critical for the development and analysis of novel antimicrobial strategies. Ultimately, this work will support the development of much needed approaches to prevent and treat costly infections.

## Methods

All chemicals are Sigma Aldrich (Dorset, UK) unless otherwise specified.

### Artificial saliva preparation

Artificial saliva was prepared by adding the following sequentially to 1 L of reverse osmosis (RO) water, stirring throughout:^69^

- ▪ 0.25 g/L sodium chloride (NaCl)
- ▪ 0.2 g/L potassium chloride (KCl)
- ▪ 0.2 g/L calcium chloride (CaCl_2_)
- ▪ 2 g/L yeast extract
- ▪ 1 g/L lab lemco powder
- ▪ 2.5 g/L hog gastric mucin (Type III, partially purified)
- ▪ 5 g/L protease peptone

The solution was stirred for 1 h at room temperature (25 °C), then autoclaved to sterilise. After autoclaving, 1.25 mL of 40% (w/v) sterile-filtered urea was added (0.22 µm filter). The artificial saliva was wrapped in foil to exclude light and prevent protein degradation. Artificial saliva was stored at 4 °C and used no later than 1 week after preparation.

### *S. sanguinis* biofilm growth

Frozen stock of *S. sanguinis* (ATCC 10556) was streaked onto a tryptone soya agar (TSA) plate and incubated at 37 °C, 5% CO_2_ for 48 h. Using the colonies grown on the agar plate, an overnight culture of *S. sanguinis* was prepared in 5 mL brain heart infusion (BHI) broth and incubated at 37 °C overnight, agitating at 100 rpm for the duration. A serial dilution in BHI broth containing 1% sucrose (w/v) was performed with the overnight culture, from 10^9^ (an optical density of ~0.5) to 10^3^ cells/mL. Individual Thermanox coverslips (Nunc, Thermo Fisher Scientific) were placed in the bottom of each well in 24-well culture plates (Nunc, Thermo Fisher Scientific). Prior to adding the planktonic culture, 1 mL of artificial saliva was added to each well containing a cover slip and left for 15 min before being removed; this was to aid initial adhesion of the bacteria. Subsequently, *S. sanguinis* monospecies biofilms were prepared by adding 1 mL of the 10^3^ dilution to each well containing a coverslip. The plates containing the biofilms were incubated for up to 7 days at 37 °C, 5% CO_2_, shaking at 100 rpm, with a change in BHI broth every 24 h, to ensure a well-established biofilm had developed. At 0, 1, 3, 5 and 7 days, analysis of biofilm growth was performed.

### Cell counting

Any remaining BHI broth from the *S. sanguinis* biofilms was removed from each well and each coverslip with biofilm was placed in 5 mL of fresh BHI broth in a universal tube. The bacteria were removed from the coverslip by sonication in an ultrasonic cleaner (In-Ceram, Vitasonic) for 10 min at 50–60 Hz, followed by agitation using a vortex mixer for 5 min. A serial dilution was performed using the Miles and Misra method to count the number of CFUs^10^. This enabled an estimation of the number of live cells found in the biofilm. To quantify the total number of cells in each biofilm, 10 µL of the lowest dilution from the serial dilution was transferred to a haemocytometer and the number of bacteria were counted in each of the corner squares.

### Fluorescent staining

For live/dead staining of *S. sanguinis* biofilms, any remaining broth was removed from each well and five coverslips were transferred to a fresh 24-well plate. A working solution of fluorescent stains was prepared by adding 3 μL of SYTO® 9 stain and 3 μL of propidium iodide stain (FilmTracer™ LIVE/DEAD® Biofilm Viability Kit, Invitrogen, USA) to 1 mL of filter-sterilised water in a foil-covered container. About 200 μL of staining solution was added onto each biofilm sample, gently so as not to disturb the biofilm. Samples were incubated for 30 min at room temperature, protected from light, before being rinsed with 200 µL filter-sterilised water. Each coverslip was then placed face up onto a clean, dry microscope slide and a drop of mounting medium added (ProLong Gold Antifade, ThermoFisher Scientific, Massachusetts, USA). A 22 mm diameter glass coverslip was used to fix the sample in place^70^. Samples were stored protected from light at room temperature (25 °C).

### Confocal laser scanning microscopy (CLSM)

Samples were imaged with CLSM (LSM 700, Zeiss, Germany) using a x40 oil immersion objective (Zeiss Objective EC Plan-Neofluar 40X/1.30 Oil DIC M27, FWD = 0.21 mm). The two stains were first imaged separately to control for any cross bleed between channels. The excitation/emission was 488 nm/<550 nm for SYTO® 9 and 555 nm/>550 nm for propidium iodide. Five random locations were scanned on each biofilm sample, resulting in 25 total images for each experimental condition. Three z-stacks were taken for each condition to calculate the biofilm thickness and for 3D visualisation and analysis. Z-stacks were taken at 1 µm increments from the surface (the first plane in which bacteria were identified).

### Image analysis

The percentage of viable and dead bacteria in each image was determined from the CLSM images. The percentage of viable bacterial was evaluated by calculating the number of pixels corresponding with the total bacteria in the image (green + red), then calculating the number of pixels corresponding with the dead (red) bacteria in the image, and finally subtracting to find the number of pixels corresponding with live bacteria. The live bacteria were quantified as a percentage of the total bacteria in each image. The image analysis method was carried out using Fiji (ImageJ, US National Institutes of Health, Bethesda, Maryland, USA) (Fig. 1). This was chosen due to it being open-source software, and therefore freely available. It should be noted that this macro calculates viability based on the assumption that the image contains a single-species biofilm, and therefore the area of red and green bacteria are proportional to the number of red and green bacteria, respectively. It is still possible to use this macro to analyse multi-species biofilms, although the output should be considered as percentage of live cell area, rather than viability.

#### Workflow

1. First, the green and red channels were separated.
2. A series of erosion, reconstruction and dilation steps were performed on each channel using a disk structuring element of size 3.
3. An additional step was applied to the red channel to compensate for the staining of extracellular DNA that can result in underestimation of the number of live cells^34^. The ‘Subtract Background’ command was applied to the red channel. This is based on a ‘rolling ball' algorithm and removes smooth continuous backgrounds from images^71^.
4. A non-linear histogram adjustment was applied to both channels using the Gamma command to correct for uneven fluorescence intensities. This allowed faint bacteria to become brighter, while the bright bacteria remained at the same intensity. The gamma value was set at 1.5.
5. The resulting images were pulled into a stack and segmented using Otsu’s threshold, with the threshold value selected based on the histogram from both images^37^.
6. The number of white pixels in the red channel was recorded from the segmented images to determine the area of dead bacteria.
7. The binary images were combined, and the total number of white pixels was recorded to determine the area of all bacteria.
8. Finally, the total area of bacteria and area of red bacteria were used to determine the percentage of viable cells. The area of all pixels can also be utilised to determine the percentage coverage of the image, which can be a useful alternative to measuring biofilm mass.

### Sensitivity and specificity analysis

A ROC curve is a performance measurement for classification problems^72^. It defines how well a model is capable of distinguishing between classes; in the current study it defined how accurate the automated process was at determining when a pixel was green or when a pixel was red. The true positive rate (TPR) or sensitivity was plotted on the y-axis and the false positive rate (FPR) or ‘1 – specificity’ was plotted on the x-axis^72^. To determine the ‘ground truth’, small sections of confocal micrographs of *S. sanguinis* biofilms were selected (three sections per image, for a total of eight images) and manually segmented in Fiji (by manually delineating all bacteria in each image) (ImageJ, US National Institutes of Health, Bethesda, Maryland, USA). The eight images included two images from each timepoint (1, 2, 5 and 7-day biofilms). The automated image analysis script was run on the 24 image sections and the resulting segmentation was compared with the ‘ground truth’ segmentation results using Eqs. 1–3:

1 $${\mathrm{TPR}}\,{\mathrm{or}}\,{\mathrm{Sensitivity}} = \frac{{{\mathrm{Number}}\,{\mathrm{of}}\,{\mathrm{true}}\,{\mathrm{positive}}\,{\mathrm{pixels}}}}{{{\mathrm{Number}}\,{\mathrm{of}}\,{\mathrm{true}}\,{\mathrm{positive}}\,{\mathrm{pixels}} + {\mathrm{Number}}\,{\mathrm{of}}\,{\mathrm{false}}\,{\mathrm{negative}}\,{\mathrm{pixels}}}}$$

2 $${\mathrm{Specificity}} = \frac{{{\mathrm{Number}}\,{\mathrm{of}}\,{\mathrm{true}}\,{\mathrm{negative}}\,{\mathrm{pixels}}}}{{{\mathrm{Number}}\,{\mathrm{of}}\,{\mathrm{true}}\,{\mathrm{negative}}\,{\mathrm{pixels}} + {\mathrm{Number}}\,{\mathrm{of}}\,{\mathrm{false}}\,{\mathrm{positive}}\,{\mathrm{pixels}}}}$$

3 $${\mathrm{FPR}} = 1 - {\mathrm{Specificity}} = \frac{{{\mathrm{Number}}\,{\mathrm{of}}\,{\mathrm{false}}\,{\mathrm{positive}}\,{\mathrm{pixels}}}}{{{\mathrm{Number}}\,{\mathrm{of}}\,{\mathrm{true}}\,{\mathrm{positive}}\,{\mathrm{pixels}} + {\mathrm{Number}}\,{\mathrm{of}}\,{\mathrm{false}}\,{\mathrm{positive}}\,{\mathrm{pixels}}}}$$

All calculations were made in MATLAB (R2018a, MathWorks Inc., USA).

### Validation of image analysis protocol on single-species biofilms

#### *P. aeruginosa* biofilm growth

A frozen stock of PA01-N was used to grow *P. aeruginosa* colonies on BHI agar at 37 °C, 5% CO_2_. Overnight cultures were grown by inoculating 5 mL of BHI broth with three colonies of PA01 and incubating at 37 °C, continuously shaking at 100 rpm for 18 h. The overnight culture was diluted using BHI broth to an optical density of 0.01 (at 600 nm), of which, 1 mL was placed in a well of a 24-well plate containing a coverslip (13 mm diameter, Thermo Scientific™ Nunc™ Thermanox™) and was performed in triplicate. The plate was then incubated for 3 h at 37 °C, shaking at 80 rpm to allow cells to adhere to the coverslip. The culture was removed from the wells and replaced with 1 mL of BHI broth, which was incubated for 24 h at 37 °C, shaking at 80 rpm. The fluorescent staining protocol was conducted as described above.

#### Multi-species *F. nucleatum* ssp. polymorphum biofilm growth

The multi-species biofilm consisted of the strains *F. nucleatum* (ATCC 10953), *A. naeslundii* (DSM 17233), *S. gordonii* (NCTC 7865) and *P. gingivalis* (W83). Overnight cultures of *F. nucleatum* were prepared in Schaedler Anaerobic broth and grown anaerobically at 37 °C. *A. naeslundii*, *P. gingivalis* and *S. gordonii* cultures were prepared in BHI broth. Bacteria were grown anaerobically at 37 °C, except *S. gordonii*, which was grown at 37 °C in 5% CO_2_. The overnight cultures were diluted with PBS (0.01 M) to an optical density of 0.5 for *S. gordonii* and 0.2 for all other species (at 600 nm). To form the biofilms, 500 µL of *A. naeslundii* and *S. gordonii* were pipetted into a well of a 24-well plate onto a coverslip (13 mm diameter, Thermo Scientific™ Nunc™ Thermanox™), and incubated with 500 µL of artificial saliva for 24 h at 37 °C. The planktonic culture was then replaced with 500 µL of *F. nucleatum* and 500 µL of artificial saliva and cultured for a further 24 h. Finally, the planktonic culture was replaced with 500 µL of *P. gingivalis* and 1.5 mL of artificial saliva. Biofilms were incubated at 37 °C until 5 days old.

#### *L. casei* biofilm growth

For the *L. casei* (NCTC 16341) biofilms, frozen stock of *L. casei* was streaked onto a De Man, Rogosa and Sharpe (MRS) agar plate and incubated at 37 °C, 5% CO_2_ for 48 h. Using the colonies grown on the agar plate, an overnight culture of *L. casei* was prepared in 10 mL MRS broth and incubated at 37 °C overnight, agitating at 100 rpm for the duration. A serial dilution in MRS broth was performed with the overnight culture, from 10^9^ to 10^3^ cells/mL. Individual Thermanox coverslips (Nunc, Thermo Fisher Scientific) were placed in the bottom of each well in 24-well culture plates (Nunc, Thermo Fisher Scientific). One microlitre of the 10^3^ dilution was added to each well containing a coverslip. The plates containing the biofilms were incubated for 7 days at 37 °C, 5% CO_2_, shaking at 100 rpm, with a change in MRS broth every 48 h. The fluorescent staining protocol was conducted as described above.

### Validation of protocol on all-dead biofilms

Five 2-day-old biofilms of *S. sanguinis* (grown as detailed above) were treated with the antimicrobial CPC (0.05% w/v) to act as a negative control for cell viability to test the image analysis protocols. One microlitre of 0.05% (w/v) CPC was added to biofilms for 5 min before fluorescent staining. As well as image analysis, a serial dilution and plating was performed to conform the viability of the antibiotic-treated biofilm. CPC treatment reduced the mean number of cells from 19 million CFU/mL to 1800 CFU/mL. Hence, the images generated under the confocal could be assumed to be 99.99% dead for the purpose of validating the image analysis protocol.

### Mouthwash study

*P. aeruginosa* (strain PA01-N) and *S. sanguinis* (ATCC 10556) were cultured overnight in Tryptone Soya broth and BHI broth, respectively. Each culture was diluted to ~10^3^ cells/mL. Individual Thermanox coverslips (Nunc, Thermo Fisher Scientific) were placed in the bottom of each well in 24-well culture plates (Nunc, Thermo Fisher Scientific). Prior to adding the planktonic culture, 1 mL of artificial saliva was added to each well containing a cover slip and left for 15 min before being removed. To grow the monospecies biofilms, 2 mL of diluted culture was added to each well and the plate was incubated at 37 °C, shaking at 40 rpm for 48 h. After incubation, the broth was removed, and the coverslips (with biofilms adhered) were placed in a clean 24-well plate. One microlitre of filter-sterilised Listerine® was used to rinse the biofilms by immersing them for 1 min. For the control group, the biofilms were rinsed with 1 mL sterile water for the same duration. The biofilms were then stained and imaged as described above.

### Biofilm formation on AM materials

#### Base material manufacturing

Ti-6Al-4V 10 x 10 x 3 mm coupons with an array of sloping angles (20, 30, 45, 60 and 90°) were fabricated with a laser powder bed fusion additive manufacture system (RenAM 500 M, Renishaw PLC, UK). A powder layer thickness of 30 μm, laser power of 200 W, a point distance of 55 μm, exposure time of 50 μs, a hatch distance of 0.105 mm and a spot size of 70–75 μm were selected^44^.

#### Bacterial colonisation assays

Bacterial colonisation on AM sample surfaces was studied by culturing *S. epidermidis* (ATCC 12228) biofilms. Samples were degreased with acetone, disinfected by autoclaving, immersed in pure ethanol for 5 min and dried under UV light. An overnight culture of *S. epidermidis* was diluted in sterile Mueller Hinton broth to a density of ~10^3^ CFU/mL and 1 mL was inoculated onto a 24-well plate containing the samples. After 24 h, all samples were moved to a new 24-well plate, washed gently three times with 10 mM phosphate buffered saline (PBS) and fixed with 2.5% (v/v) glutaraldehyde in PBS for 1 h^44^.

#### Bacterial imaging

One sample for each sloping angle was washed gently three times with 10 mM PBS. Samples were stained with 200 μL of SYTO® 9 and propidium iodide solution (FilmTracer™ LIVE/DEAD® Biofilm Viability Kit, Invitrogen, USA) and incubated for 30 min. Imaging was carried out using a Zeiss LSM 710 confocal microscope (Carl Zeiss GmbH, Germany) at x10 magnification^44^.

### Statistical analysis

All statistical analyses were conducted in GraphPad Prism (v. 5.03). For the mouthwash study, a paired *t*-test was conducted to compare the two conditions: mouthwash-treated and water-treated. For the AM material study, a Kruskal–Wallis test (one-way ANOVA) was used to determine any significant differences between the sloping angles. For all analyses, *p* < 0.05 was considered statistically significant.

### Reporting Summary

Further information on research design is available in the Nature Research Reporting Summary linked to this article.

## Supplementary information

Supplementary Information

Reporting Summary

## Data Availability

The datasets used and/or analysed during the current study are available from the corresponding author on reasonable request.

## Code availability

The macro is available from: https://github.com/sophie-mountcastle/Biofilm-Viability-Checker/.
