# Supplementary material for: Biofilm viability checker: An open-source tool for automated biofilm viability analysis from confocal microscopy images
Source: NPJ Biofilms Microbiomes. 2021 May 14;7:44. doi: 10.1038/s41522-021-00214-7 (PMC8121819; doi:10.1038/s41522-021-00214-7)

Supplementary Figures

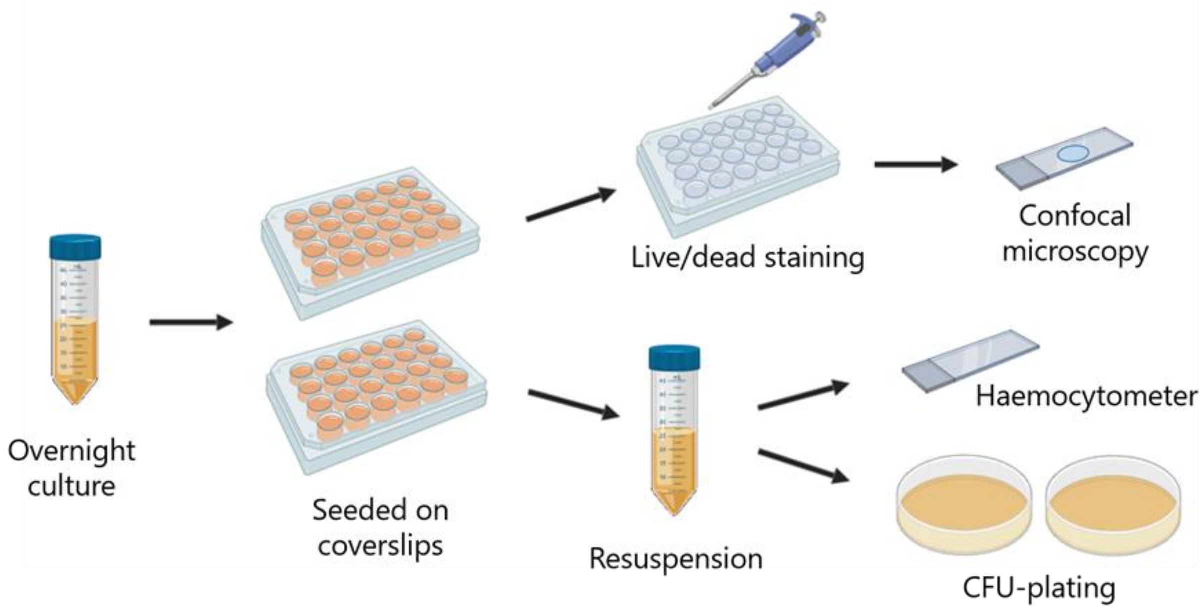

Supplementary Figure 1: Summary of method used to grow S. sanguinis biofilms and compare image analysis and traditional microbiological quantification methods. An overnight culture of S. sanguinis was diluted down to approximately 10^3 cells/mL and seeded on coverslips in a 24-well plate. At each time point (0, 1, 2, 5 & 7 days), one plate was sacrificed, stained using a fluorescent live/dead stain and imaged using confocal microscopy. Subsequent image analysis was used to determine the percentage of live cells at each time point. The remaining biofilms were re-suspended in fresh media using sonication and vortexing to remove them from the coverslip. From these suspensions, the total number of live cells was established using CFU-plating. Total bacteria (live and dead) were counted using a haemocytometer. We acknowledge the use of BioRender® (BioRender.com) to create Supplementary Figure 1.

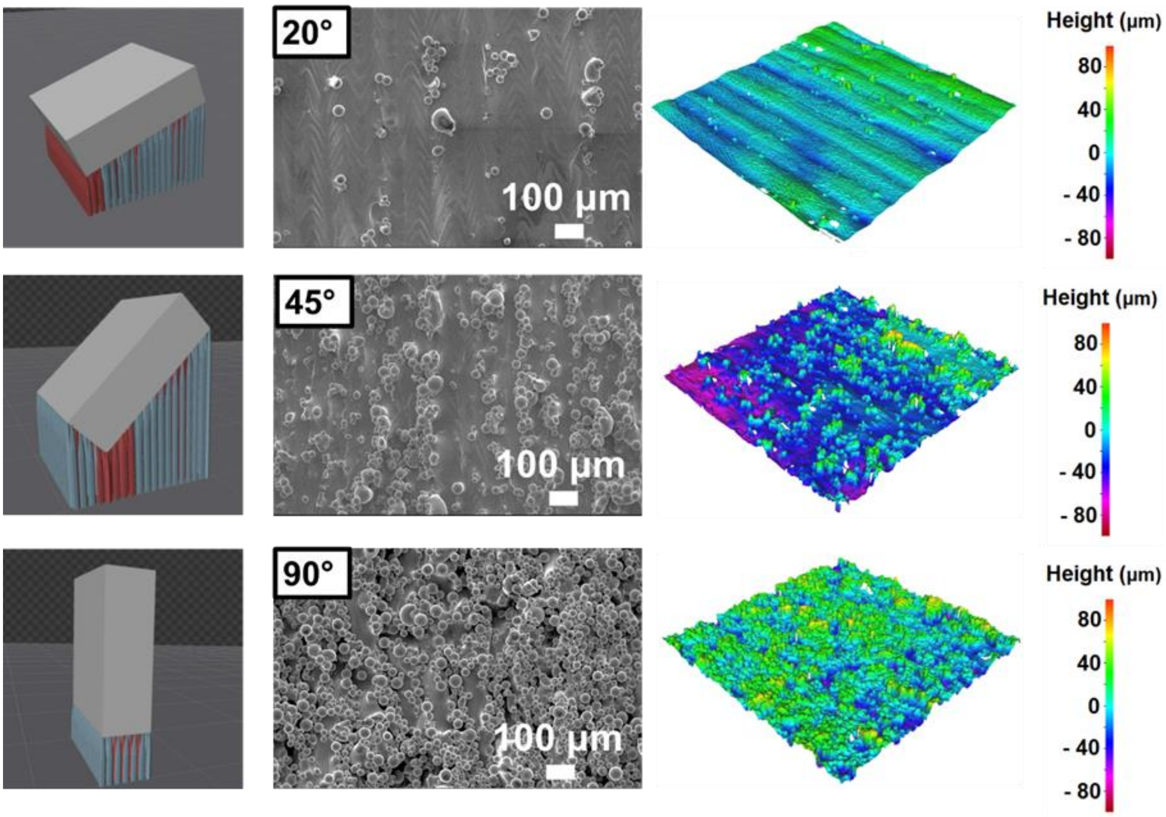

Supplementary Figure 2: 3D model of sample orientation and supported surfaces, SEM micrograph images and topographic scan of as-built Ti-6Al-4V coupons with sloping angles 20 - 90°. Figure reproduced with permission from Villapun et al. (2020) "A design approach to facilitate selective attachment of bacteria and mammalian cells to additively manufactured implants". Additive Manufacturing 36:101528.

Supplementary Information

The macro written to implement the automated image analysis described in the main manuscript is available from https://github.com/sophie-mountcastle/Biofilm-Viability-Checker/. It can be opened in ImageJ by saving the .ijm file in the desired location and subsequently dragging it to the ImageJ toolbar:

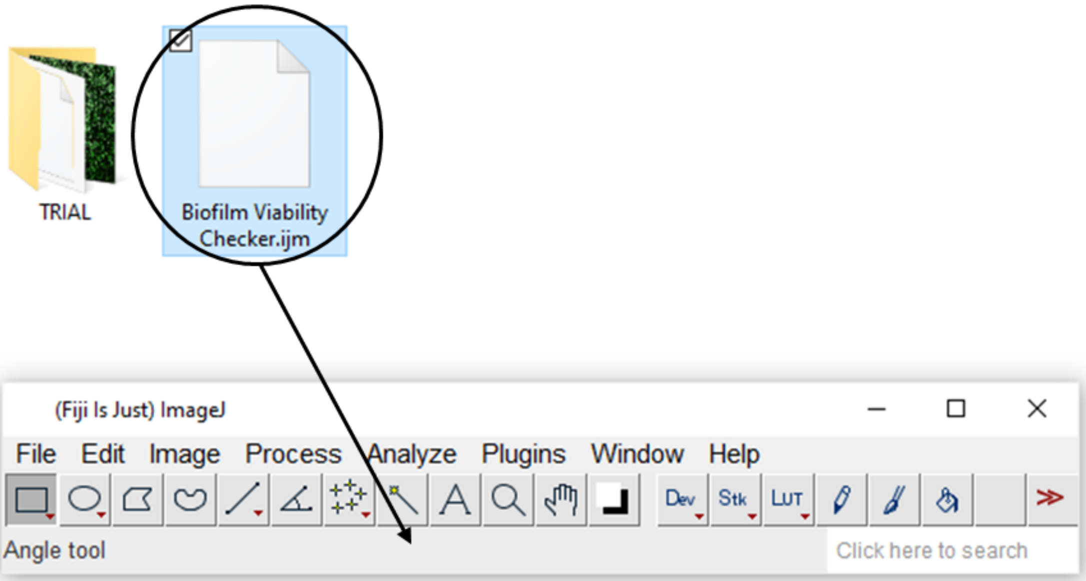

Please note that the Fiji distribution is required to run the code. It is open-source and can be downloaded for free from: https://imagej.net/Fiji/Downloads. In addition, the MorphoLibJ (https://imagej.net/MorphoLibJ) plugin needs to be installed for the macro to operate. This can be done by following the instructions provided on the ImageJ website here: https://imagej.net/MorphoLibJ.html#Installation (for more in depth installation support, see: https://imagej.net/Following_an_update_site). We recommend running these macros on TIFF files.

The macro calculates and outputs the total number of fluorescent pixels (green and red) and total number of red pixels for a series of images saved within one folder. All image windows are closed after the macro is completed. It can also output an outline of the segmented regions

superimposed on the original image. This is useful when assessing the effect if any changes are made to the protocol, for example adjusting the structuring element size or removing pre-processing steps.

Instructions on how to implement the macros are as follows:

1. Open the Fiji distribution of ImageJ.

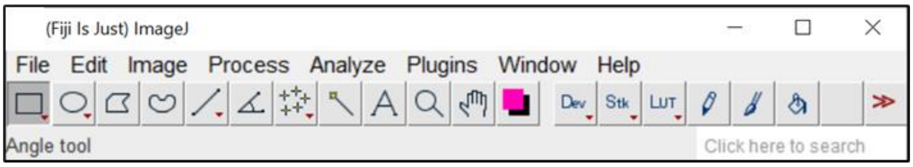

2. Drag the macro file to the ImageJ toolbar to open it.

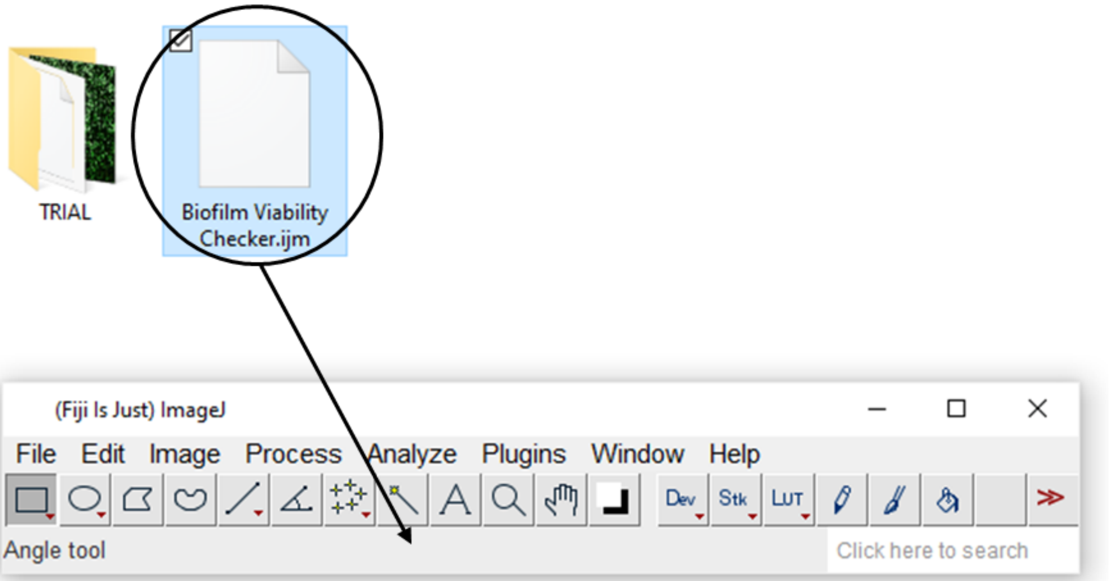

3. The macro will open in a new window.

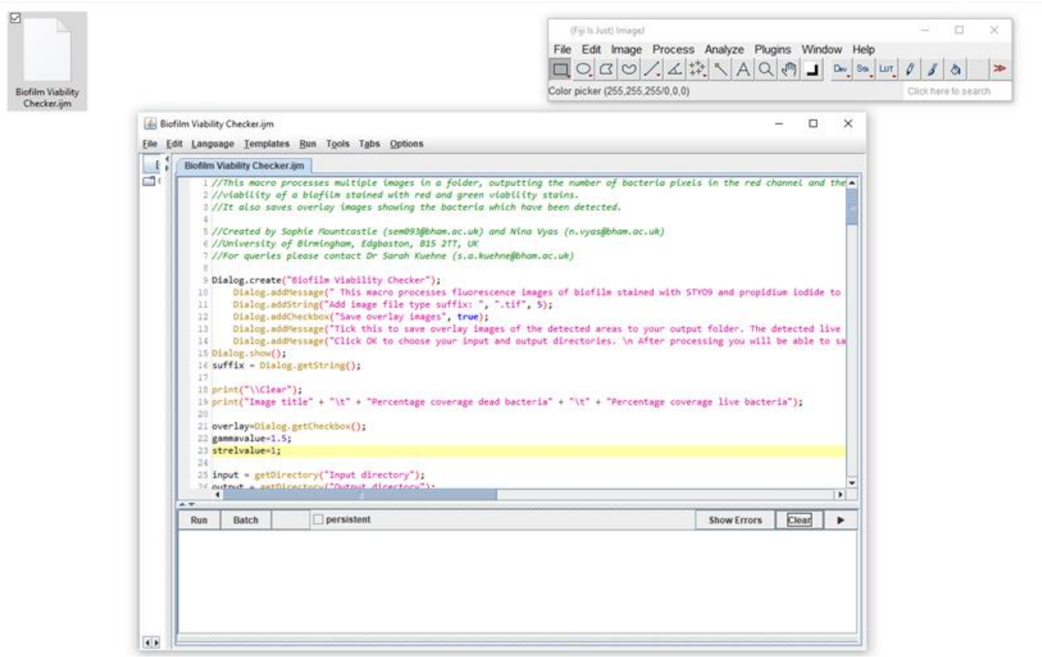

4. Click “Run”.

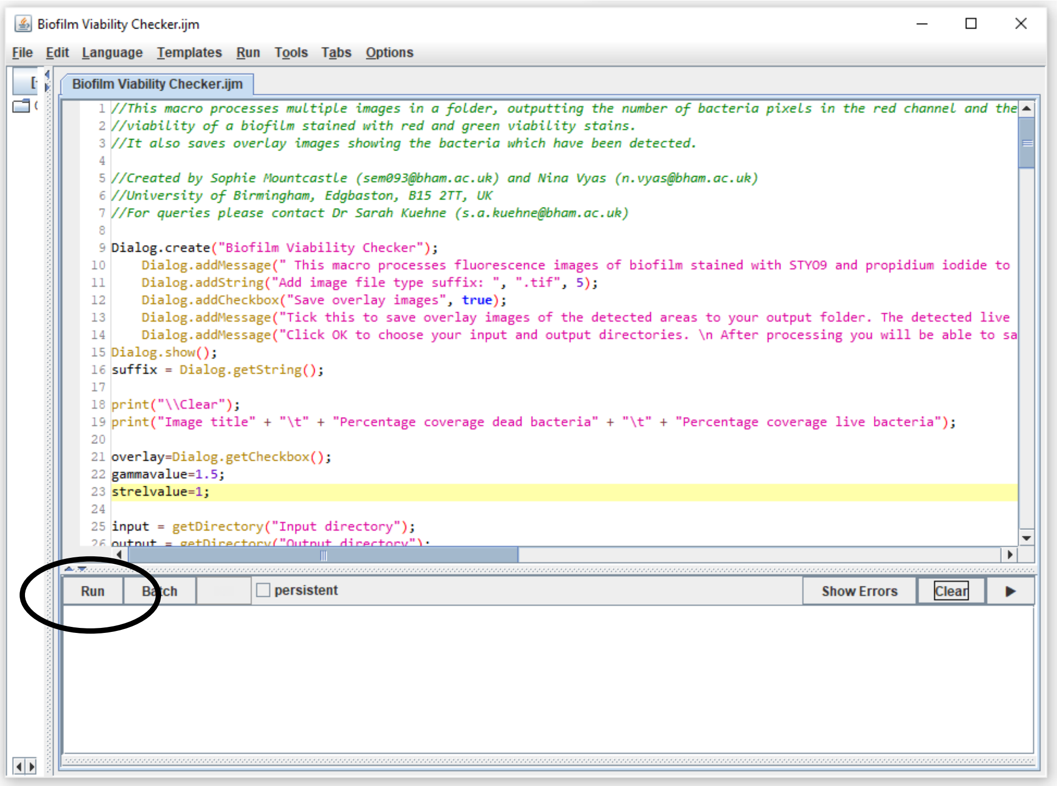

5. A new window will pop up with instructions for how to operate the macro.

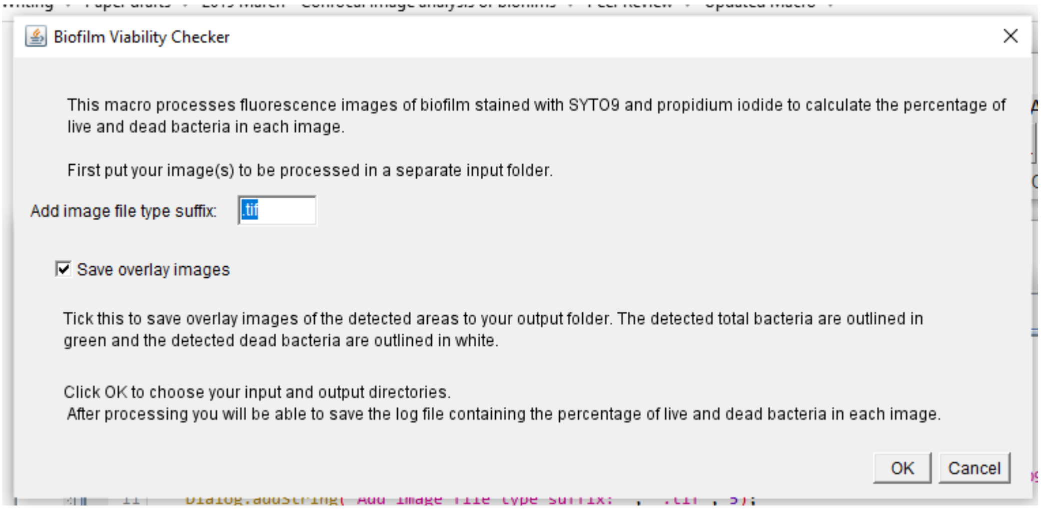

6. Follow the instructions by inputting the file suffix (we suggest TIFF files work best, but this can be amended).

7. If you wish for the macro to output the images with an overlay of the red and total bacteria outlines, tick the box next to "Save overlay images". Untick the box if you only want the results file to be saved.

8. Click OK and a new window will open asking for an Input Directory to be selected. Navigate to the folder containing the images you wish to analyse and click "Select".

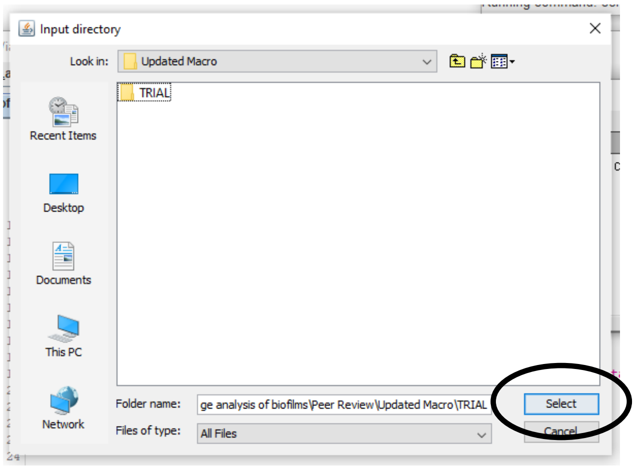

The following image is a sample of the expected input confocal micrograph for this macro. Please note that scale bars affect the result and plain images should be used without a scale bar included.

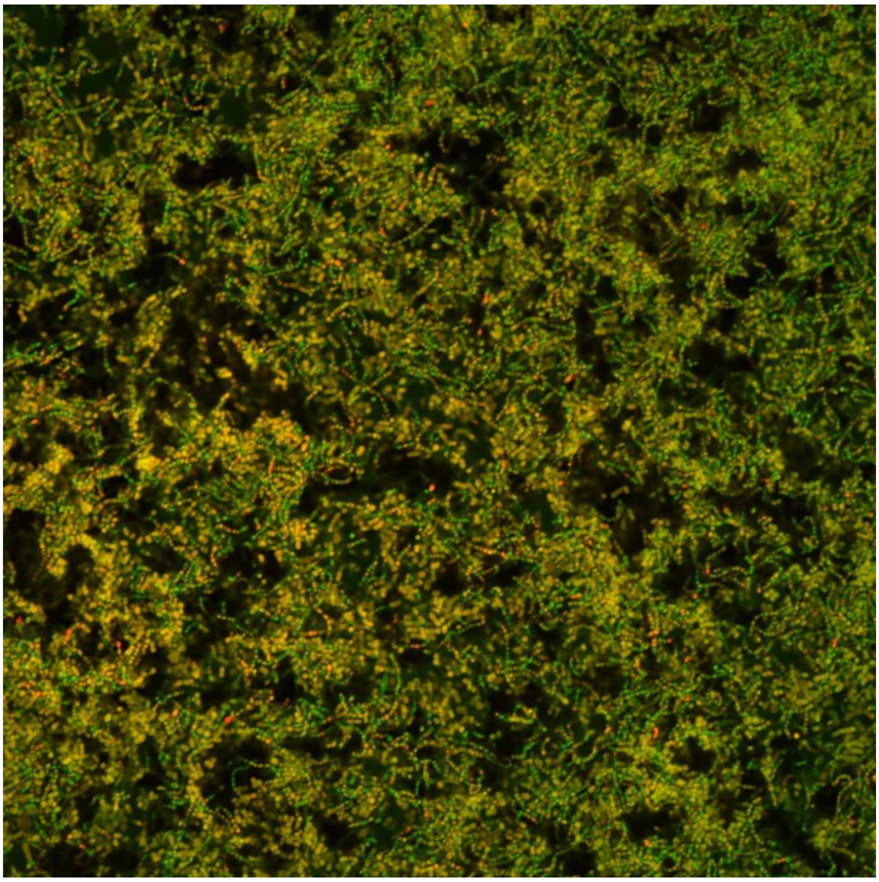

Sample image: Five-day biofilm of S. sanguinis stained with the FilmTracer Live/Dead Biofilm Viability Kit.

9. Next, the macro will ask you for the Output Directory. This is where the results of the image analysis will be saved, along with the outlined overlay images if that option has been selected. Please note: the output directory cannot be the same as the input directory. Click “Select”

10. Once the macro has calculated the results, it will ask you to choose a name for the results file. Enter the chosen file name and click “Save”.

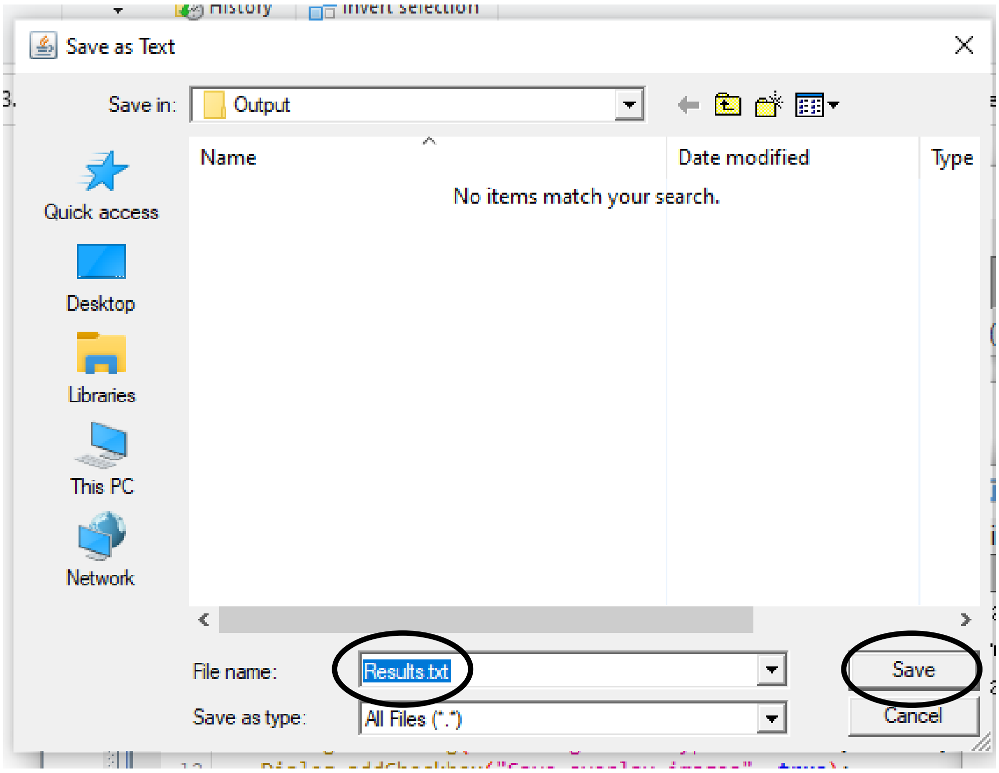

11. Navigate to the log file in the File Explorer. The log file provides the image name, the percentage of dead bacteria, and percentage of live bacteria (percentage viability). The results open as a .txt file in Notepad but can be copy and pasted directly into Excel for analysis.

The following image is an example of the outlined image output from the macro, as well as the results of the analysis of the sample image.

Image title	Percentage of dead bacteria	Percentage of live bacteria (viability)
5day_sample4_image1.tif	54.5	45.5

98

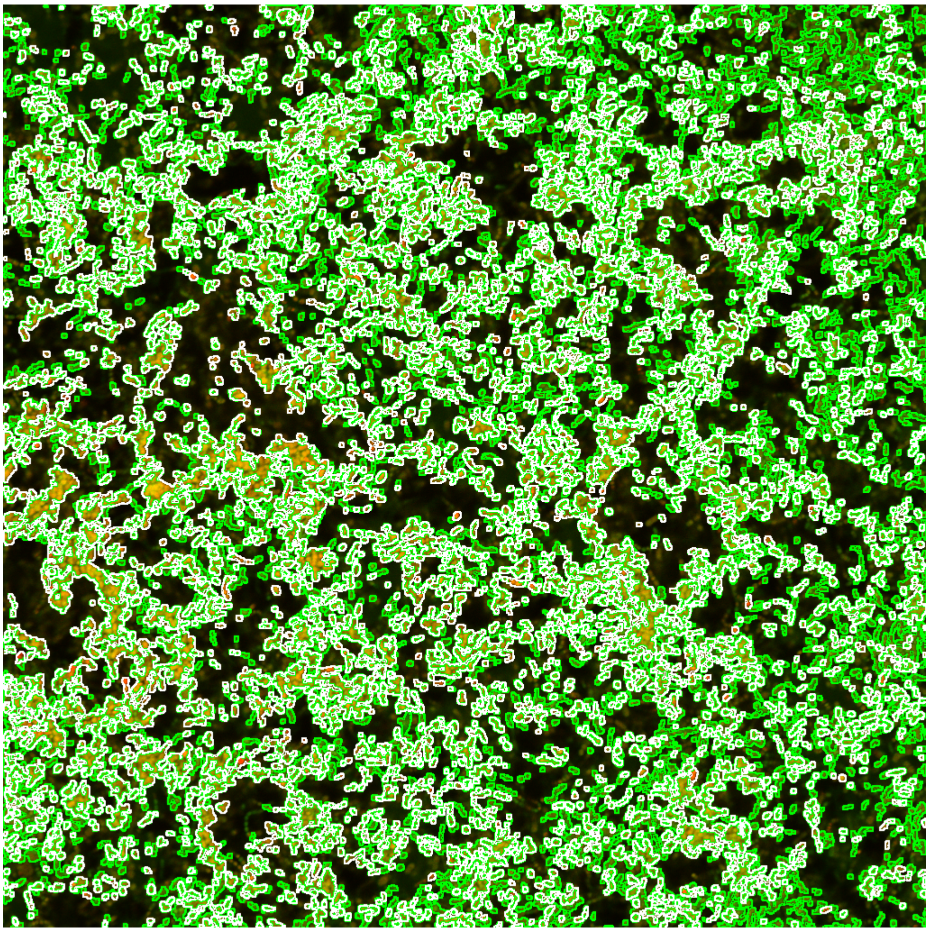

Supplement: Supplementary file 1 — Supplementary Information [file 41522_2021_214_MOESM1_ESM.pdf]
